# Factors influencing nursing performance of long-term care hospital nurses in Korea

**DOI:** 10.1093/geroni/igaf122.3025

**Published:** 2025-12-31

**Authors:** So Young Shin, Shin Young Park

**Affiliations:** Inje University, Busan, Busan, Republic of Korea; Inje University, Busan, Busan, Republic of Korea

## Abstract

**Purpose:**

The purpose of this study is to identify the effect of resilience, communication competence, nursing work environment on nursing performance of nurses at long-term care hospitals in Korea.

**Methods:**

Among staff nurses at 5 long-term care hospitals located in B metropolitan city in Korea, 137 subjects who have a long-term care experience of more than 1 year participated in this study. Data collection was conducted with the structured self-report questionnaire survey. The collected data were analyzed with descriptive statistics, independent t-test and one-way ANOVA, Scheffé test for post-hoc analysis, Pearson’s correlation coefficient and multiple linear regression.

**Results:**

The mean age of 137 subjects was 50.96(±10.17) years. Nursing work performance was positively correlated with resilience (r=.70, p<.001), communication competence (r=.59, p<.001), and nursing work environment (r=.23, p=.007). In the multiple linear regression analysis, factors influencing nursing performance of subjects were resilience (β=.54, p<.001) and communication competence (β=.19, p=.037). The explanatory power of resilience and communication competence on nursing performance was 51% (F = 24.13, p<.001).

**Conclusion:**

The results from this study show that for long-term care hospital nurses, resilience and communication competence have a significant effect on their nursing performance. To enhance nursing performance of nurses at long-term care hospitals, it is necessary to develop effective mediating programs specifically improving resilience and communication competence.

